# *5-HTTLPR* Genotype Moderates the Effects of Past Ecstasy Use on Verbal Memory Performance in Adolescent and Emerging Adults: A Pilot Study

**DOI:** 10.1371/journal.pone.0134708

**Published:** 2015-07-31

**Authors:** Natasha E. Wright, Judith A. Strong, Erika R. Gilbart, Skyler G. Shollenbarger, Krista M. Lisdahl

**Affiliations:** 1 Department of Psychology, University of Wisconsin-Milwaukee, Milwaukee, WI, United States of America; 2 Department of Anesthesiology, University of Cincinnati, Cincinnati, OH, United States of America; University of Chicago, UNITED STATES

## Abstract

**Objective:**

Ecstasy use is associated with memory deficits. Serotonin transporter gene (*5-HTTLPR*) polymorphisms have been linked with memory function in healthy samples. The present pilot study investigated the influence of *5-HTTLPR* polymorphisms on memory performance in ecstasy users, marijuana-using controls, and non-drug-using controls, after a minimum of 7 days of abstinence.

**Method:**

Data were collected from 116 young adults (18–25 years-old), including 45 controls, 42 marijuana users, and 29 ecstasy users, and were balanced for *5-HTTLPR* genotype. Participants were abstinent seven days prior to completing memory testing. Three MANCOVAs and one ANCOVA were run to examine whether drug group, *5-HTTLPR* genotype, and their interactions predicted verbal and visual memory after controlling for gender, past year alcohol use, other drug use, and nicotine cotinine levels.

**Results:**

MANCOVA and ANCOVA analysis revealed a significant interaction between drug group and genotype (*p* = .03) such that ecstasy users with the L/L genotype performed significantly worse on CVLT-2 total recall (*p* = .05), short (*p* = .008) and long delay free recall (*p* = .01), and recognition (*p* = .006), with the reverse pattern found in controls. Ecstasy did not significantly predict visual memory. *5-HTTLPR* genotype significantly predicted memory for faces (p = .02); short allele carriers performed better than those with L/L genotype.

**Conclusions:**

*5-HTTLPR* genotype moderated the effects of ecstasy on verbal memory, with L/L carriers performing worse compared to controls. Future research should continue to examine individual differences in ecstasy’s impact on neurocognitive performance as well as relationships with neuronal structure. Additional screening and prevention efforts focused on adolescents and emerging adults are necessary to prevent ecstasy consumption.

## Introduction

The lifetime prevalence rate of ecstasy (MDMA, or 3,4-methylenedioxymethamphetamine) use among adolescents is 11.3% [1], with girls reporting more use [2]. Neurocognitive developmental changes are abundant in adolescence and last into emerging adulthood [3], with changes in the prefrontal cortex and the limbic system leading to increased vulnerability to risky behavior and the neurotoxic effects of drug use (see [4]). Some suggest that due to this malleable time period of brain development, later life events may not be able to correct for inadequate development during teen years [5]. Therefore, it is important to understand the neurocognitive impact of ecstasy use, and to help determine at-risk groups who may experience poorer outcomes.

Exposure to MDMA, the major component in ecstasy or Molly, impacts reuptake of serotonin (5-HT), and to a lesser extent norepinephrine and dopamine [6–8]. MDMA has been implicated in loss of serotonin in rats and humans [8–15]. This may be due, in part, to the ability of MDMA to reverse serotonin transporters through stimulating serotonin efflux, resulting in an overabundance of serotonin being released at once [16], as well as doubling the amount of 5-HTT expressed several hours after MDMA consumption [8]. In animal studies, MDMA has been shown to be particularly neurotoxic to the serotonergic system [8–10, 13]. Serotonergic recovery has been shown with at least a year of abstinence, though the axons exhibited alternate patterns of functioning compared to controls [9], even after 7 years in squirrel monkeys [10]. Serotonin neurotoxicity has also been demonstrated in humans; in a positron emission tomography (PET) study of ecstasy users, Kish and colleagues [11] found decreased serotonin transporter binding throughout the cortex and specifically in the hippocampus in comparison to controls, even after a minimum of 45 days abstinence. Decreased binding was further predictive of worse verbal memory performance. Other PET studies have found similar reductions in 5-HT binding across cortical sites [12] and availability, including in the hippocampus [17]. Therefore, converging lines of evidence have suggested that the hippocampus may be particularly vulnerable to ecstasy-related damage [11, 18–22]. Consistent with this hypothesis, adolescent ecstasy users show abnormal functional activation of the hippocampus during a verbal working memory task [23]. While there may be some recovery of 5-HT neurons with abstinence [24], Ando and colleagues [25] found that the hippocampus and thalamus recovered less than the rest of the cortex.

Consistent with these hippocampal abnormalities, four meta-analyses have concluded that ecstasy exposure is linked with reduced verbal memory, with medium to large effect sizes [26–29]. For example, Kalechstein and colleagues [27] found deficits in attention, verbal and nonverbal memory and learning, motor speed, and executive functioning systems in ecstasy users, with the largest effect size on verbal learning and memory (Cohen’s *d* = .73) and nonverbal learning and memory the next largest (Cohen’s *d* = .58). More specifically, with two exceptions [30–31], studies have found verbal learning and memory deficits in ecstasy users compared to controls [20, 28, 32–37]. Ecstasy users also self-report more memory deficits in comparison to non-ecstasy drug users and controls [38]. However, at least two studies only found memory deficits within the context of polydrug use, not specific to ecstasy consumption [39, 40]. Results regarding visual memory are more limited, with fewer studies investigating how it is affected by ecstasy exposure, though those that have found mixed results. Rosier and colleagues [41] found no significant differences in visual memory performance among ecstasy users when compared to controls, however, several other studies have found visual memory deficits related to ecstasy exposure [19, 32, 42].

Inconsistencies in the literature on memory function in ecstasy users may be explained by individual differences in genes that code for serotonin transporters, as there has been minimal investigation into the potential moderating capacity of serotonin transporter gene (*5-HTTLPR*). In particular, *5-HTTLPR* has been associated with memory function (*5-HTTLPR*) [11, 43]. The short (S) allele of *5-HTTLPR* has decreased reuptake of serotonin transporter (SERT, or 5-HTT) compared to the long (L) allele, perhaps due to having 40% fewer SERT binding sites [44]. L/L genotype carriers also have higher densities of 5-HTT as well as increased 5-HTT expression in comparison to S carriers [8, 45]. Due to downregulation, this may result in overall reduced 5-HT signaling [46], and the S allele has been linked with increased risk for affective disorders [47] and greater depressive symptoms [48]. However, at least one study has found that in the presence of a stressor (family discord), those with the L/L 5-HTTLPR genotype demonstrated significantly higher rates of anxiety [49].

In healthy controls, we found that the S allele was associated with larger left hippocampal volumes, poorer verbal memory and greater depressive symptoms in females, demonstrating a gender specific functional disadvantage [43]. The L/L genotype has also been found to be protective against postpartum depression symptoms [50], while S carriers with chronic stress have had increased depression symptoms in young adults at-risk for depression [48]. However, only a few studies have examined the interaction between *5-HTTLPR* genotype and ecstasy use. In ecstasy users, carriers of the S/S allele have exhibited deficits in verbal fluency [51] as well as decreased cardiovascular and more sedative subjective effects following consumption [52]. S carrier ecstasy users have also been found to have increased risk for mood disorder symptoms [53]. In regards to memory, *5-HTTLPR* genotype moderated the effects of ecstasy on visual memory [41–42]. Cuyas and colleagues [42] found that lifetime ecstasy use negatively correlated with visual memory, and users with *5-HTTLPR* S alleles exhibited poorer performance. Rosier and colleagues [41] similarly found in an adult sample that S carriers trended towards significantly worse visual memory and decision making than healthy controls. However, other studies have found that in the presence of depression, the L allele may confer a functional disadvantage [54–55]. Ecstasy users with L/L genotype have also been found to have greater changes in 5-HTT gene expression, though these results are absent of full statistical analyses due to limited power [8]. Therefore, there is preliminary evidence that the *5-HTTLPR* genotype may moderate the effects of ecstasy on cognition.

As ecstasy users rarely only use ecstasy, marijuana use co-morbidity is a common issue in studying the unique effects of ecstasy [56]. The presence of executive functioning deficits that are not accounted for by polydrug use is debated [39, 42, 57], though the two drugs have been shown to interact with gender in glial and serotonergic cells [58] and several studies have found deficits in ecstasy users that were above and beyond that of controls and marijuana users [20, 36, 42, 59–60]. Independently, marijuana has been associated with memory deficits [39, 61–65]. There is also some evidence that marijuana may interact with *5-HTTLPR*. The short allele has been shown to be more closely related to bipolar disorder diagnosis in marijuana users [66], and marijuana users with S/S genotype performed worse on a risk-taking task compared to S/S controls, though verbal memory was not assessed [67]. Therefore, there appears to be some overlap between the effects of ecstasy and marijuana on memory function and the two substances may uniquely interact with 5-*HTTLPR*, necessitating the inclusion of a marijuana control group.

The present pilot study was conducted to investigate the independent and interactive influences of ecstasy use and *5-HTTLPR* polymorphisms on memory functioning, while controlling for comorbid marijuana use. It was predicted that ecstasy use and the *5-HTTLPR* genotype would interact in predicting verbal and visual memory impairments, with S carriers demonstrating the greatest memory deficits in comparison to healthy controls and L/L genotypes [41–43].

## Materials and Methods

### Participants

One-hundred sixteen participants were recruited through fliers and advertisements in community newspapers. Forty-four controls, 43 marijuana users, and 29 ecstasy users, were balanced for *5-HTTLPR* genotype (28 S carrier controls, 28 S carrier marijuana users, 17 S carrier ecstasy users), fitting with larger population estimates of the distribution of S carriers to L/L genotype in mostly white samples [68]. Seventy-two participants (62%) were white. Nine (31%) of the ecstasy users met DSM IV criteria for ecstasy dependence. Exclusion criteria included co-morbid independent Axis I disorders, major medical or neurologic disorders, prenatal issues (e.g., gestation > 35 weeks) or prenatal exposure to alcohol (>4 drinks/day or >7 drinks/week) or illicit drugs (>10 uses), and excessive other-drug use (>50 uses of any drug category except nicotine, alcohol, marijuana, and ecstasy in lifetime). Control subjects had fewer than 3 lifetime ecstasy uses and 10 lifetime marijuana uses. Marijuana group subjects had fewer than 6 ecstasy uses and had used marijuana more than 50 times lifetime. Ecstasy users were so grouped if they had reported more than 10 lifetime ecstasy uses during initial screening; upon more detailed collection of substance use history, it was revealed that not all participants met this initial inclusion criterion and therefore those in the ecstasy group have a minimum of 7 lifetime uses. Participants were required to remain abstinent from alcohol or drug use for seven days prior to the study session.

### Procedure

The University of Cincinnati Institutional Review Board approved all aspects of this study. Advertisements were placed in newspapers and on college campuses to recruit participants. Interested participants called in and, after providing oral consent, completed a phone screen using a semi-structured interview to assess for Axis I disorders [69]. If eligible and after obtaining informed written consent, eligible participants completed the parent imaging genetics study (PI: Lisdahl, 1R03 DA027457) in either one or two sessions. Those with substance use histories completed the psychological questionnaires, drug use interview, neuropsychological battery, and MRI scan in two sessions (typically 2–3 days apart). Those with minimal use completed the study in one session. Participants were paid $160 for two sessions ($110 for one) and received parking reimbursement, local substance treatment resources and images of their brain.

#### Biological samples

Participants underwent a urine toxicology screen using the One Step Drug Screen Test, a breathalyzer test, and female participants were administered a pregnancy test. Those who tested positive for drugs and/or alcohol except cannabis and nicotine were excluded. Metabolite levels of THC were examined for participants that tested positive for marijuana using mass spectrometry testing; session 2 THC:creatinine metabolite ratios were subtracted from session 1 total ratios to ensure there were no increases or current use during the week of memory testing (see [70]).

#### Drug use

Ecstasy and other drug use was measured using the Timeline Follow-Back (TLFB). A modified version of the *Time-Line Follow-Back* [69, 71] technique was used, which utilizes memory cues of common holidays and personal events to measure frequency of drug use over the past year (assessed month-by-month for one year). Additionally, a semi-structured interview was administered to measure frequency/quantity of lifetime drug use [69]. For each drug category, participants were asked their average weekly use for each year of use. The following drug categories were assessed: ecstasy or Molly, alcohol, marijuana, sedatives (e.g., downers, ketamine, GHB), stimulants (amphetamine, methamphetamine, cocaine, crack cocaine), hallucinogens (mushrooms, PCP, LSD, peyote), opioids (heroin, opium), and inhalants (nitrous oxide, paint, glue, household cleaners, gas). The participant’s drug use was measured in standard units (tablets for ecstasy; standard drinks for alcohol; joints for marijuana).

#### Memory

The California Verbal Learning Test—2^nd^ edition (CVLT-2) [72] was used to measure verbal memory. Participants are read 20 words belonging to four semantic categories and asked to recall as many as possible across five learning trials. They are then read a 20-word distracter list. After a short delay, they are asked to recall the first list given cues about semantic categories. After a 20-minute delay, free and cued recall, and recognition ability are measured. The Rey Osterrieth Complex Figure Test (RCFT) [73] and the Faces I and Faces II subtests from the Wechsler Memory Scale-III (WMS-III) [74] were used to assess visual memory. For the RCFT, participants first copied a unique picture containing various shapes and markings, then drew the figure again from memory immediately and after a delay. For the WMS-III Faces tasks, during the initial presentation, participants were exposed to a series of 24 target faces. Immediately following, participants were shown a second series of 48 faces (24 old, 24 new) and asked to recognize the target faces (Faces I). After a delay, participants were shown another series of 48 faces and were again asked to identify the target faces that were from the initial presentation (Faces II).

#### Genotyping

As described in Price et al. [43], Dr. Judith Strong performed the genotyping for 5*-HTTLPR* L/S promoter polymorphism [75]. The amplification products were digested with restriction enzyme HpaII in order to analyze the functional A/G SNP (rs25531) within the L/S promoter polymorphism. L alleles with G at the rs 25531 site were scored as S alleles. Details of the amplification method and primers used were as in Thompson et al. [76].

#### Data analysis

ANOVAS and chi-squares were run to examine potential demographic differences between drug groups and genotype. To assess the independent and interactive effects of ecstasy use and *5-HTTLPR* status on verbal and visual memory, MANCOVAs were run to examine whether groups (controls, MJ users, and ecstasy users) differed on 1) verbal memory (total recall, short delay free recall, and long delay free recall), 2) visual figure memory (immediate, delayed recall), and 3) visual faces memory (immediate, delayed recall). All memory variables included in each of the three MANCOVAs were significantly correlated within their own domains (i.e., verbal memory, object memory, and faces memory; r’s>.70). All variables were normally distributed, except for verbal memory recognition. Therefore, after logarithmically transforming the variable, we ran verbal memory recognition as a separate ANCOVA. We did not adjust for multiple comparisons as this is a small pilot study and such a correction would increase the likelihood of Type II error too significantly. Covariates included gender, cotinine levels (nicotine metabolite), past year alcohol use (total number of standard drinks), and total other drug use (all episodes combined). Because nicotine and alcohol use have previously been shown to influence verbal memory performance [77, 78] and due to the polydrug use which frequently occurs with MDMA use, cotinine, past year alcohol use, and other drug use were chosen as covariates, though they were not found to influence results in the present study.

## Results

### Demographics

Drug groups did not differ significantly on gender [x^2^ = 2.5, p = .3], age [F(2,113) = 2.9, p = .06], ethnicity [x^2^ = 9.7, p = .5], education [F(2,113) = 1.0, p = .4], reading ability [F(2,113) = 1.0, p = .4], income [F(2,113) = 0.7, p = .5], serotonin genotype [x^2^ = 0.5, p = .8], or past year cigarette use [F(2,113) = 2.9, p = .06] (see Table 1). As genetic results are often influenced by ethnicity [79], we confirmed our findings through running the same analyses using only white subjects; results remained unchanged. Drug groups differed on recent nicotine use (cotinine level) [F(2,112) = 14.3, p < .001], past year ecstasy [F(2,113) = 13.8, p < .001], alcohol [F(2,113) = 8.2, p < .001], marijuana [F(2,113) = 9.3, p < .001], and other drug use (including ecstasy) [F(2,106) = 10.87, p < .001]; cotinine, alcohol, and other drug use are controlled for in the MANCOVA analysis (see Table 1). Post-hoc analysis revealed that the ecstasy and marijuana groups both differed from controls, but not from each other, in their past year alcohol, marijuana, and nicotine use (*p*’s>.10). Ecstasy users reported significantly more other drug use, especially hallucinogens, although this was limited to no more than 25 times in their lifetime.

**Table 1:** Demographics by Drug Group and Genotype.

	Controls (n = 44)		MJ (n = 43)		Ecstasy (n = 29)	
	% or M (SD) Range		% or M (SD) Range		% or M (SD) Range	
	S carrier	L/L	S carrier	L/L	S carrier	L/L
*5-HTTLPR* Genotype	28	16	28	15	17	12
	(64%)	(36%)	(65%)	(35%)	(59%)	(41%)
Age	21.3	20.5	21.3	20.8	22.2	22.2
	(2.2)	(2.3)	(2.3)	(2.4)	(2.1)	(2.2)
	18–25	18–25	18–25	18–25	18–25	19–25
Education	14.1	13.3	13.2	13.9	13.6	13.0
	(1.8)	(1.4)	(1.8)	(2.2)	(2.0)	(1.6)
	11–18	11–16	9–17	12–19	11–18	11–17
Reading Score	97.8	104.4	102.9	103.7	100.1	97.4
	(10.4)	(10.1)	(14.5)	(15.9)	(10.6)	(13.3)
	78–120	89–120	73–133	80–134	82–115	67–112
Gender (% female)	50%	56%	32%	47%	35%	33%
% White	57%	56%	71%	53%	71%	58%
* ^a^ Past Year Ecstasy Use	0	0	0.1	0.1	36.9	14.3
	(0)	(0)	(0.3)	(0.5)	(62.3)	(12.0)
			0–1	0–2	0–255	1–41
* ^a^ Lifetime Ecstasy Use (tablets)	0	0	0.5 (1.2)	0.8	448	303.8
	(0)	(0)	0–5	(1.7)	(1062.5)	(672.3)
				0–6	7–4026	11–2202
* ^b^ Past Year Marijuana Use (joints)	.3	.8	468.6	406.2	845.4	1025.3
	(1.4)	(1.7)	(830.0)	(410)	(1731.3)	(1334.4)
	0–7	0–5	10–3895	4–1662	0–7343	12–4179
* ^b^ Past Year Alcohol Use (standard drinks)	69.5	95.3	293.0	360.5	394.2	179.2
	(79.4)	(212.8)	(288.9)	(482.1)	(435.1)	(243.7)
	0–320	0–878	0–914	7–1724	0–1426	0–884
* ^b^ Cotinine Level	1.2	1.9	4.0	3.6	3.8	4.3
	(1.7)	(2.7)	(2.3)	(2.6)	(2.4)	(2.3)
	0–6	0–6	0–6	0–6	0–6	1–6
* ^a^ Other Drug Use	0.04	0.3	11.0	2.3	111.1	159.7
	(0.2)	(0.8)	(32.5)	(3.1)	(198.7)	(302.3)
	0–1	0–3	0–170	0–10	0–827	0–1038

Notes: M = mean; SD = standard deviation.

*p < .05

a = ecstasy users significantly greater than MJ users and controls

b = MJ users and ecstasy users significantly greater than controls

Genotype groups also did not differ on gender [*x* ^*2*^ = .55, *p* = 0.46], age [F(1,114) = 1.1, p = .3], ethnicity [*x* ^*2*^ = 4.66, *p* = 0.46], education [F(1,114) = 0.4, p = .5], reading ability [F(1,114) = 0.6, p = .4], income [F(1,114) = 0.02, p = .9], or past year alcohol [F(1,114) = 0.1, p = .8], marijuana [F(1,114) = 0.1, p = .8], nicotine [F(1,114) = 1.8, p = .2], and other drugs [F(1,114) = 0.4, p = .5]. Further, when looking only at the ecstasy group, the two genotype groups did not differ on gender [*x* ^*2*^ = .01, *p* = 0.6], age [F(1,27) = .01, p = .9], ethnicity [*x* ^*2*^ = 2.5, *p* = 0.5], education [F(1,27) = 0.7, p = .4], reading ability [F(1,27) = 0.4, p = .6], income [F(1,27) = 0.6, p = .4], or past year alcohol [F(1,27) = 2.4, p = .13], marijuana [F(1,27) = 0.1, p = .8], nicotine [F(1,27) = 2.4, p = .14], and other drugs [F(1,27) = 0.3, p = .6], past year [F(1,27) = 1.5, p = .2] or lifetime [F(1,27) = .2, p = .7] ecstasy use, nor did they differ on length of abstinence from ecstasy [F(1,25) = 0.01, p = .9].

### Frequency/Quantity of substance use

In ecstasy users, mean lifetime ecstasy use was 388.3 tablets (SD = 909.9; range 7–4026), with a mean use of 27.5 tablets (SD = 49.0; range 0–255) in the past year. Ecstasy users also used marijuana an average of 919.8 joints in the past year (SD = 1555.8; range 0–7343). In marijuana using controls, mean lifetime ecstasy use was 0.6 tablets (SD = 1.4). Marijuana users’ mean lifetime marijuana use was 1450.1 joints (SD = 3136.4; range 10–17821), with a mean use of 446.8 joints (SD = 706.9) in the past year. All participants demonstrated negative toxicology tests for all drugs, including MDMA, and reduced levels of THC from session one to two, demonstrating at least one week of abstinence from all drugs. The ecstasy users were abstinent from ecstasy for an average of 75 days (SD = 71; range 7–274).

### Verbal memory

#### Ecstasy Use

**Fig 1:**
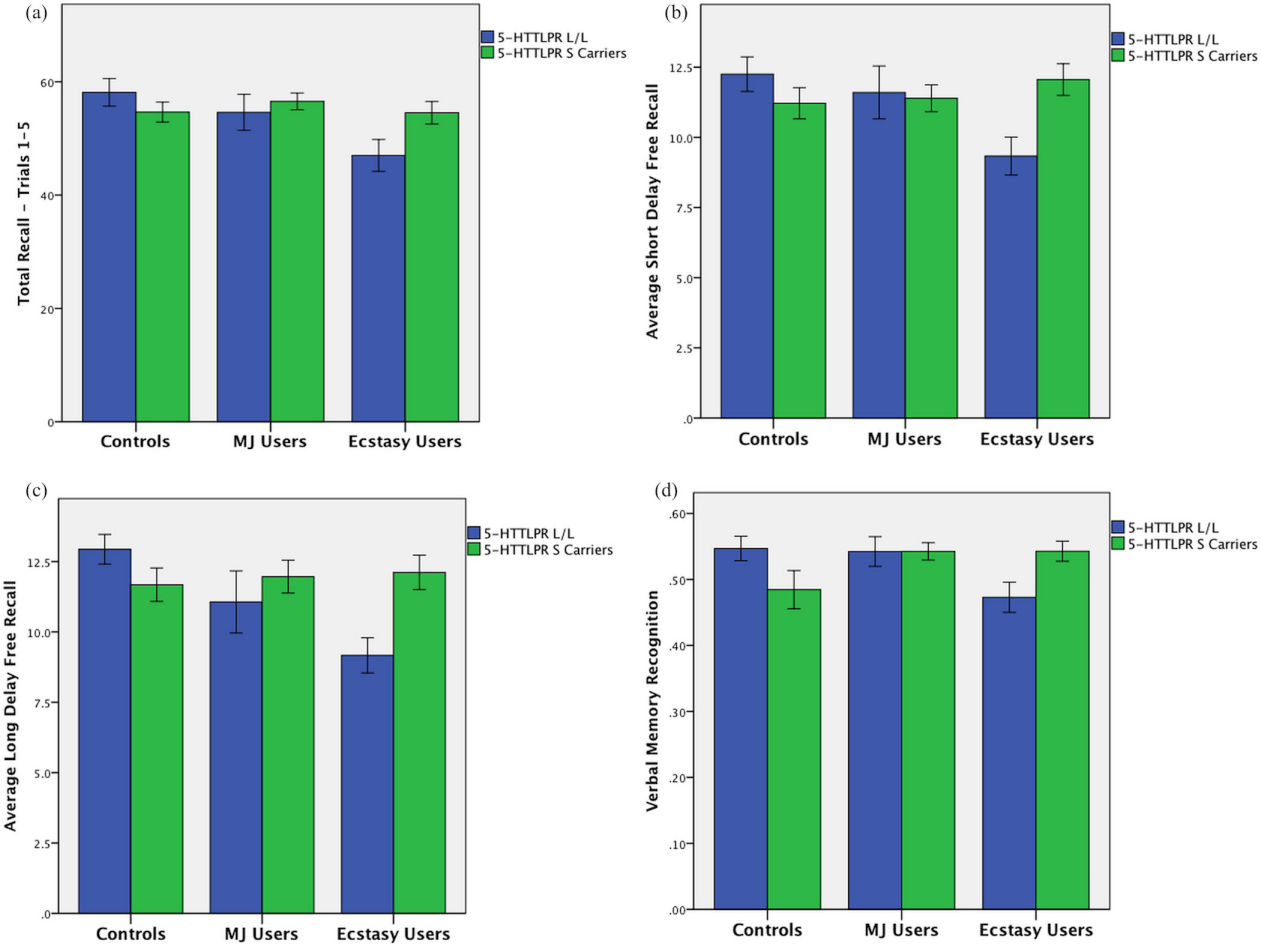
Average Verbal Memory Performance According to Drug Group and 5-HTTLPR Genotype. *5-HTTLPR* genotype interacted with ecstasy group to predict (a) total recall of the first 5 trials; (b) average short delay free recall; (c) average long delay (20 minute) verbal recall; and (d) average recognition. In the non-using controls, the S allele was associated with poorer verbal memory while in the ecstasy users, the L/L genotypes performed worse than S carriers and both control groups.

MANCOVA analysis revealed that after controlling for recent nicotine, alcohol and other drug use, ecstasy group status did not independently predict verbal memory. ANCOVA analysis of verbal memory recognition revealed that after controlling for recent nicotine, alcohol and other drug use, ecstasy group status did not independently predict verbal memory. Genotype. Similarly, genotype did not independently predict verbal memory performance. Ecstasy*5-HTTLPR Genotype. A significant interaction between ecstasy group status and *5-HTTLPR* genotype [Pillai’s trace = .12, F(6,206) = 2.28, p = .04] was observed in predicting CVLT-2 verbal memory. Post-hoc analysis revealed that *5-HTTLPR* genotype interacted with ecstasy group to predict total recall [F(2,104) = 2.99, p = .05], short delay free recall [F(2,104) = 5.27, p = .007], long delay free recall [F(2,104) = 4.88, p = .009; see Fig 1]. Similarly, in examining the ANCOVA analysis, ecstasy group interacted with *5-HTTLPR* genotype in predicting total recognition discriminability [F(2,104) = 4.73, p = .01; see Fig 1]. In the non-using controls, the S allele was associated with poorer verbal memory in each domain while in the ecstasy users, the L/L genotypes performed worse than S carriers and both control groups.

### Visual memory

Ecstasy Use. MANCOVA analysis revealed that after controlling for recent nicotine, alcohol and other drug use, ecstasy group status did not independently predict RCFT visual memory. Genotype. *5-HTTLPR* genotype predicted WMS Faces performance [Pillai’s Trace = .08, F(2,103) = 4.3, p = .01]. Post-hoc analysis revealed that *5-HTTLPR* genotype predicted Faces total recall I [F(1,104) = 6.2, p = .01] and II [F(1,104) = 8.7, p = .004]. Examination of estimated marginal means (see Table 2) revealed that S carriers performed better on both Faces I and II recall compared to L/L genotype carriers. Ecstasy*5-HTTLPR Genotype. No significant interactions were revealed between ecstasy group status and *5-HTTLPR* genotype.

**Table 2:** Estimated Marginal Means for Verbal and Visual Memory Performance by Drug Group and Genotype.

	Controls		MJ		Ecstasy	
	*M* (SE)		*M* (SE)		*M* (SE)	
Verbal Memory	S carrier	L/L	S carrier	L/L	S carrier	L/L
Immediate Total Raw	54.7 (2.0)	58.7 (2.4)	57.2 (1.9)	54.7 (2.5)	54.4 (2.4)	47.2 (3.0)
Short Delay	11.2 (.6)	12.4 (.7)	11.8 (.5)	11.8 (.7)	12.0 (.7)	9.0 (.8)
Long Delay	11.5 (.6)	13.0 (.8)	12.3 (.6)	11.3 (.8)	12.2 (.8)	9.0 (1.0)
Recognition	.48 (.020)	.55 (.024)	.55 (.019)	.55 (.025)	.55 (.025)	.47 (.030)
Visual Memory						
RCFT Immediate Recall	19.4 (1.3)	20.1 (1.6)	20.4 (1.3)	20.5 (1.6)	18.6 (1.6)	19.8 (1.9)
RCFT Delayed Recall	18.6 (1.2)	18.9 (1.5)	19.5 (1.2)	20.7 (1.5)	18.1 (1.5)	19.5 (1.8)
WMS Faces I	37.4 (.9)	37.2 (1.0)	38.5 (1.0)	38.4 (.9)	40.6 (1.3)	37.4 (1.1)
WMS Faces II	39.1 (.8)	37.5 (.9)	38.5 (.9)	38.3 (.8)	41.3 (1.2)	37.7 (.9)

Notes: *M* = mean; SE = standard error

## Discussion

The aims of the current study were to examine the independent and interactive effects of ecstasy (MDMA) exposure and *5-HTTLPR* genotype on verbal and visual memory in abstinent adolescents and emerging adults. The findings demonstrated that *5-HTTLPR* status moderated the impact of ecstasy use on verbal memory function in youth. As hypothesized, in controls, the *5-HTTLPR* S allele was associated with poorer verbal memory. However, ecstasy and marijuana users demonstrated the opposite pattern, the *5-HTTLPR* L/L genotype was associated with inferior verbal memory performance. Results further revealed that *5-HTTLPR* genotype predicted memory for faces; S carriers performed superior to individuals with the L/L genotype.

This study lends further evidence that ecstasy users demonstrate significant verbal memory deficits in new learning, retention, and recognition of verbal material with relatively intact visual memory [11, 20, 32–33, 35–37, 57], but only in L/L ecstasy users. The current sample of ecstasy-users used an average of 27 ecstasy tablets in the past year (80% using 25 or fewer tablets in the past year), demonstrating that compared to other drugs of abuse, relatively low levels of exposure may result in verbal memory deficits. For example, marijuana studies include participants who use marijuana several times a week to multiple times a day, resulting in hundreds of uses over a year. Further, consistent with our prior study [20], the ecstasy users were abstinent from ecstasy for an average of 2.5 months (minimum 7 days), suggesting that verbal memory deficits may continue despite significant periods of abstinence. These converging lines of evidence warrant consistent screening for ecstasy consumption in schools and universities with clear warnings that even recreational use of ecstasy may result in significant verbal memory problems, particularly in those with specific genotypes (here, L/L serotonin transporter genotype).

We previously [43] reported that in healthy controls, females with the S allele had poorer verbal memory, larger hippocampal volumes, and greater depressive symptoms compared to the L/L genotype, suggesting that the S allele may be associated with reduced 5-HT signaling in healthy samples. In support of this, the current study demonstrated that non-drug using individuals with the S allele performed more poorly on verbal memory tasks. This supports the previous studies demonstrating that the S allele was associated with poorer downstream serotonin signaling [44], which may place one at risk for poorer verbal memory or increased risk for serotonin-related psychiatric disorders [47]. The current study also identified a significant at-risk group for negative outcomes; those who had the L/L serotonin transporter genotype and used ecstasy recreationally demonstrated the most robust verbal memory deficits. This is in contrast with our original hypothesis, based on prior findings [41, 53], that the S allele would confer a functional disadvantage in the drug users. The current findings suggest that the downstream functional relationship between *5-HTTLPR* genotype and memory may be different in drug users, or the drugs directly impacted the serotonin system, altering the functional consequence of the genotype. This may also be due to the increased 5-HTT gene expression in ecstasy users with the L/L genotype, compared to S carrier ecstasy users [8], which may result in greater downstream downregulation of the serotonin system following repeated use. Similar findings have been reported in depression [54–55] and those with significant family discord [49]. For example, individuals with late-onset depression and the *5-HTTLPR* L/L genotype demonstrated significantly smaller hippocampal volumes while those with early-onset depression demonstrated the opposite pattern. On balance, Frodl and colleagues [54] found reduced gray matter volumes in PFC and limbic regions, including the hippocampus in *5-HTTLPR* S allele carriers in controls, while the opposite was found in depressed patients. In summary, it is possible that damaging a robustly active serotonin system may result in increased functional consequences, or drugs such as ecstasy interact with the *5-HTTLPR* genotype in a unique manner [8]. Converging evidence suggests a link between *5-HTTLPR* genotype, hippocampal structure and memory, although this relationship may be moderated by the presence of a serotonin stressor (i.e., mood disorders, family discord, or ecstasy exposure). Additional research is needed to examine the impact of ecstasy and marijuana on hippocampal serotonin signaling across *5-HTTLPR* genotypes in youth.

Similar to Rosier et al. [41] and Medina et al. [20], the current study did not find significant visual memory deficits in ecstasy users. Also, this study found a functional advantage to the *5-HTTLPR* S allele in predicting facial memory performance. Similarly, Rosier and colleagues found that independent of ecstasy use, volunteers with the S/S genotype performed better than those with the L/L genotype on a visual planning task. In addition, Anderson and colleagues [80] found that in a healthy sample S carriers performed better on a task of visual working memory. Perhaps, then, if the L/L allele is related to increased serotonin signaling, then this increased signaling may be related to improved verbal memory but worse visual memory in otherwise healthy individuals. These findings provide preliminary evidence suggesting a role for the *5-HTTPLR* genotype in visual memory for faces and calls into question the idea that the S allele is always a risk allele. This finding needs to be confirmed with additional studies focused on visual processing and memory.

Replication in science is important. Candidate gene studies in particular tend to have greater issues in replicability [81]. Importantly, *5-HTTLPR* has been associated with serotonin diseases. For example, an analysis of the *5-HTTLPR* haplotype utilizing genome-wide association study (GWAS) data (n = 1505 cases and 2168 controls) found that the S allele of *5-HTTLPR* is associated with major depressive disorder [82]. However, a recent GWAS mega-analysis examining genetic predictors of MDD did not reveal significant findings, which was attributable to poor power [83]. Therefore, additional GWAS and candidate gene studies are needed to examine the downstream endophenotypes associated with the *5-HTTLPR* gene in healthy and serotonin-stressed subgroups (e.g., ecstasy users, those with MDD) [84]. Future studies should seek to replicate this particular endophenotypic finding.

The present findings are limited by a few important factors. First, due to low sample sizes we were unable to examine whether gender or ethnicity moderate the relationships between *5-HTTLPR* genotype and memory function [36, 68]; either gender or ethnicity may interact with *5-HTTLPR* genotype in predicting memory function and should be assessed in future studies. Second, a common issue in ecstasy studies is polydrug use. While we controlled for other drug use, alcohol, and nicotine use and included a marijuana control group, it is still difficult to assess the unique effects of ecstasy used *simultaneously* with other drugs. Third, while we had a fairly large sample, we did not have enough power to separately assess L/S or S/S genotype. This may obscure some of the potential differences between polymorphisms. Other genes also may have a unique influence on memory due to interactions with the serotonin system, such as *COMT* [51] or *BDNF* [85]. Larger datasets that combine imaging genetics data and drug use information are needed to examine potential genetic epistasis. Replication of these results is also necessary; we were unable to replicate this study within an independent sample in the present paper due to logistical issues in recruiting new participants, but this will be an important step in future studies. Due to the skewed distribution of use, we were unable to assess the potential dose-dependent relationship between drug use and memory with genotype. Finally, we did not test for the purity of ecstasy or Molly, and therefore other substances may have been included in the pills (most often caffeine and other stimulants); however, reports from online testing in the United States reports show that the majority of ecstasy or Molly tablets do contain MDMA [86], with purity even higher in non-domestic ecstasy tablets [87].

## Conclusions

In summary, the present study investigated the independent and interactive effects of ecstasy use and serotonin transporter gene (*5-HTTLPR*) on verbal and visual memory. We found that 5-*HTTLPR* genotype significantly moderated ecstasy’s impact on verbal memory. While in controls the L allele tends to be a protective factor, it appears to be a risk factor in ecstasy poly-drug users. These findings are particularly interesting when considering the novelty of this study and the level of difficulty in recruiting this population. Further, results demonstrate that even recreational ecstasy use is associated with verbal memory deficits, particularly in those with the *5-HTTLPR* L allele. This may be especially true in developing adolescents and emerging adults. Scientists developing large, longitudinal, multi-site imaging genetics studies are encouraged to consider the impact of genetics, and ecstasy exposure on the developing brain, as well as assessing the influence of gender.

## Supporting Information

S1 File Dataset. (XLSX)
